# Knottin cyclization: impact on structure and dynamics

**DOI:** 10.1186/1472-6807-8-54

**Published:** 2008-12-12

**Authors:** Annie Heitz, Olga Avrutina, Dung Le-Nguyen, Ulf Diederichsen, Jean-François Hernandez, Jérôme Gracy, Harald Kolmar, Laurent Chiche

**Affiliations:** 1 CNRS, UMR5048, Université Montpellier 1 et 2, Centre de Biochimie Structurale, 34090 Montpellier, France; 2 INSERM, UMR554, 34090 Montpellier, France; 3 Institute for Organic and Biomolecular Chemistry, Georg-August University, Göttingen, Germany; 4 Clemens-Schöpf Institute of Organic Chemistry and Biochemistry, University of Technology, Darmstadt, Germany; 5 CNRS, FRE3009, SysDiag, 34093 Montpellier, France; 6 CNRS, UMR5247, 34093 Montpellier, France

## Abstract

**Background:**

Present in various species, the knottins (also referred to as inhibitor cystine knots) constitute a group of extremely stable miniproteins with a plethora of biological activities. Owing to their small size and their high stability, knottins are considered as excellent leads or scaffolds in drug design. Two knottin families contain macrocyclic compounds, namely the cyclotides and the squash inhibitors. The cyclotide family nearly exclusively contains head-to-tail cyclized members. On the other hand, the squash family predominantly contains linear members. Head-to-tail cyclization is intuitively expected to improve bioactivities by increasing stability and lowering flexibility as well as sensitivity to proteolytic attack.

**Results:**

In this paper, we report data on solution structure, thermal stability, and flexibility as inferred from NMR experiments and molecular dynamics simulations of a linear squash inhibitor EETI-II, a circular squash inhibitor MCoTI-II, and a linear analog lin-MCoTI. Strikingly, the head-to-tail linker in cyclic MCoTI-II is by far the most flexible region of all three compounds. Moreover, we show that cyclic and linear squash inhibitors do not display large differences in structure or flexibility in standard conditions, raising the question as to why few squash inhibitors have evolved into cyclic compounds. The simulations revealed however that the cyclization increases resistance to high temperatures by limiting structure unfolding.

**Conclusion:**

In this work, we show that, in contrast to what could have been intuitively expected, cyclization of squash inhibitors does not provide clear stability or flexibility modification. Overall, our results suggest that, for squash inhibitors in standard conditions, the circularization impact might come from incorporation of an additional loop sequence, that can contribute to the miniprotein specificity and affinity, rather than from an increase in conformational rigidity or protein stability. Unfolding simulations showed however that cyclization is a stabilizing factor in strongly denaturing conditions. This information should be useful if one wants to use the squash inhibitor scaffold in drug design.

## Background

The knottins are fascinating miniproteins present in many species and featuring various biological actions such as toxic, inhibitory, antimicrobial, insecticidal, cytotoxic, anti-HIV, or hormone-like activities [1]. They share a unique knotted topology of three disulfide bridges, with one disulfide penetrating through a macrocycle formed by the two other disulfides and interconnecting peptide backbones. The KNOTTIN database  provides standardized data on sequences, structures and other information on known knottins, also referred to as "inhibitor cystine knot" (ICK) proteins [2,3]. The main knottin features are a remarkable stability due to the cystine knot, a small size making them readily accessible to chemical synthesis, and an excellent tolerance to sequence variations. Knottins therefore appear as appealing leads or scaffolds for peptide drug design [1,4-8]. The knottin scaffold is found in almost 30 different protein families among which conotoxins, spider toxins, squash inhibitors, agouti-related proteins and plant cyclotides are the most populated families. Cyclotides are knottins from plants in the *Rubiaceae *and *Violaceae *families that, until recently, were always shown to be head-to-tail macrocyclic peptides [9-11]. In contrast, all known structurally similar squash inhibitors were linear knottins [12]. This difference held many years until the discovery of the first macrocyclic squash inhibitors *Momordica cochinchinensis *trypsin inhibitor (MCoTI)-I and -II [13], and, more recently, of a linear cyclotide [14]. The oxidative folding of squash inhibitors and cyclotides has been thoroughly studied [15-20]. In both cases, the folding has been shown to occur via a two-disulfide intermediate whose structure is very native-like. This intermediate is the direct precursor of the three-disulfide knotted miniprotein for the squash inhibitors but not for the cyclotide kalata B1. It is now clear that both cyclic and linear variants can exist in different knottin families, but the reasons for this, and the impact of the cyclization, are still poorly understood. Macrocyclic peptides are expected to display improved stability, better resistance to proteases, and reduced flexibility when compared to their linear counterparts, hopefully resulting in enhanced biological activities. On the other hand, although peptide, and, more specifically, knottin cyclization has been shown to be accessible to chemical synthesis [21] and biosynthesis [22], the cost for the cyclization should not be neglected in view of potential pharmaceutical applications of the knottin scaffold. It is thus of interest to carefully evaluate the role and importance of the cyclization in the different knottin families. Despite several studies, the impact of the cyclization in the cyclotide series is still unclear since linearization of kalata B1 was shown to eliminate hemolytic activity [23], whereas a naturally occurring linear cyclotide displayed a reduced but not suppressed hemolytic activity [14]. In both cases, the structures were essentially conserved but higher flexibilities of the linear compounds were described. However, no large differences in thermal and enzymatic stability were described between kalata B1 and acyclic permutants [24]. On the other hand, very little is known on the difference between linear and cyclic squash inhibitors beside the fact that they share similar 3D structures [25,26]. Nevertheless, it has been stated recently that the rigidifying cyclic backbone of MCoTI-II contributes enhanced stability in comparison to the simpler linear squash inhibitors, prompting the development of improved methods for cyclic knottin production [27].

**Figure 1:**
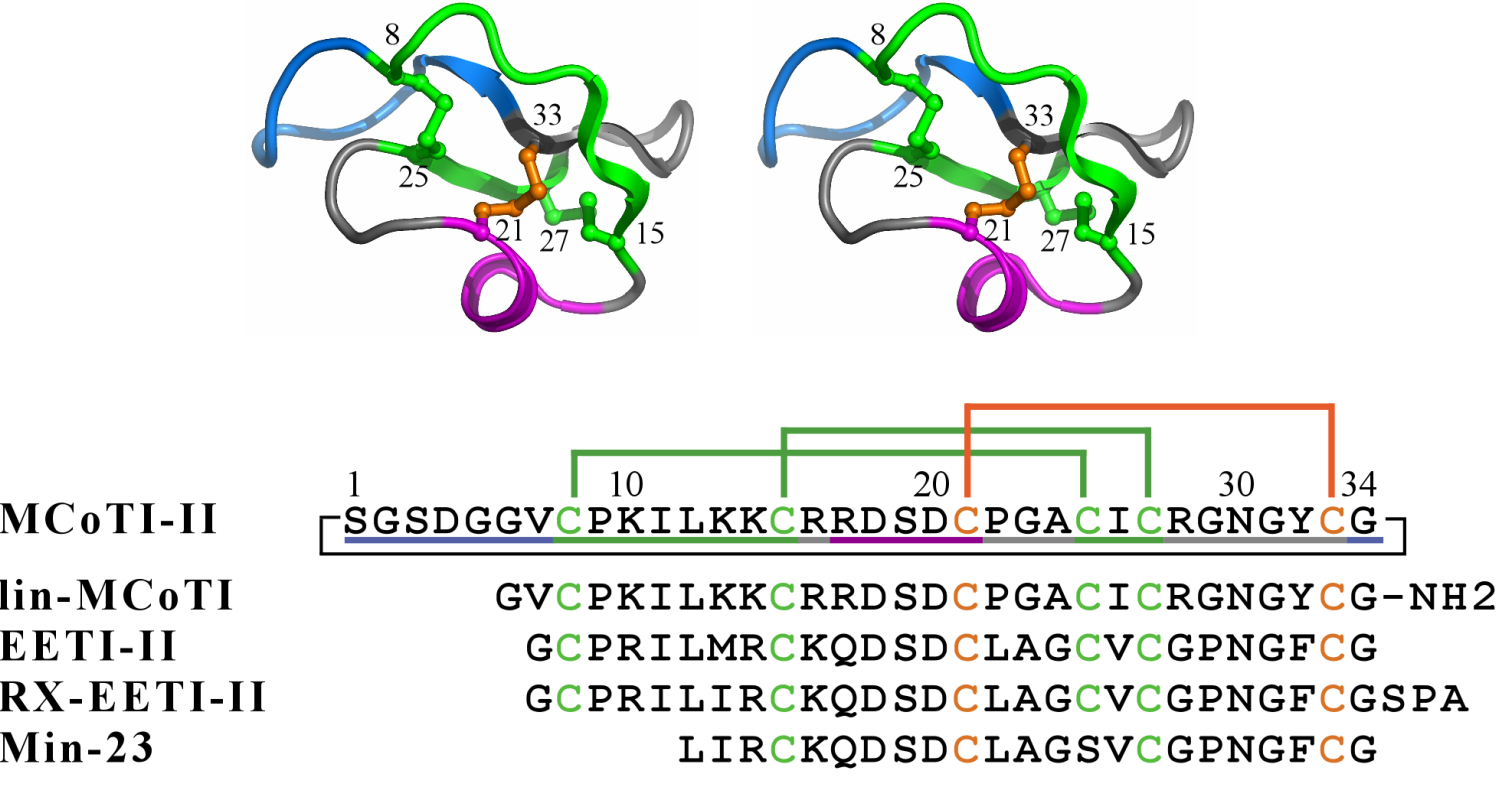
**The knottin fold**. (Top) Stereoscopic view of a schematic representation of MCoTI-II, a head-to-tail cyclized squash inhibitor. The head-to-tail linker is shown in blue. Disulfide bridges are shown as ball-and-stick representations. The cystine knot is shown in green (the disulfide macrocycle) and orange (the penetrating disulfide). *β*-strands are shown as flat arrows and the 3_10_-helix turn is shown in magenta. Cysteines are numbered. (Bottom) Sequences of the squash inhibitors used in this work. Numbering follows MCoTI-II. The disulfide bridge coloring scheme follows the one used in the structure. The colors used in the three-dimensional structure are shown using a colored line below the MCoTI-II sequence. Peptide cyclization is displayed as a black line for MCoTI-II.

In this study, we report on structure, stability, and dynamics of cyclic and linear squash inhibitors and analogs. Figure 1 shows the scaffold and the amino acid sequences of the cyclic squash inhibitor, MCoTI-II, of the synthetic linear variant used in this work, lin-MCoTI, and of the naturally occurring linear squash inhibitor, *Ecballium elaterium *trypsin inhibitor (EETI)-II. The solution structure of the linear analog of MCoTI-II, lin_MCoTI and a refined solution structure of EETI-II were first determined and compared to the MCoTI-II structure. These structures were then used to evaluate the impact of the cyclization on the flexibility and on the stability through NMR experiments, molecular dynamics simulations, and thermal unfolding simulations. We show that, in contrast to what could have been intuitively predicted and has been explicitly stated [27], the head-to-tail cyclization has minimal impact on structure and flexibility of MCoTI-II in standard conditions, possibly explaining why most squash inhibitors are linear. This finding raises the question as to why few squash inhibitors have evolved into cyclic compounds. However, although all compounds are remarkably stable and rigid at room temperature, we also show that the cyclic compound can endure heat better than the linear ones in high temperature simulations.

## Results

### Solution structures of lin-MCoTI and of EETI-II

To compare solution structures of free linear squash inhibitors to the solution structure of the cyclic squash inhibitor MCoTI-II, we selected wild type EETI-II and the synthetic linear analog of MCoTI-II, i.e. lin-MCoTI. As the solution structure of EETI-II currently available in the Protein Data Bank [28] (PDB ID: 2eti) was determined in 1989 using NMR data from 360 MHz spectra and corresponds to a crude distance geometry structure without molecular mechanics refinement [29,30], a new refined EETI-II solution structure was determined. NMR spectra of EETI-II and of lin-MCoTI were therefore recorded to determine their three-dimensional structures. The sequences are displayed in Figure 1 along with the numbering scheme used in this study that follows the MCoTI-II sequence with numbers from 1 to 34.

**Figure 2:**
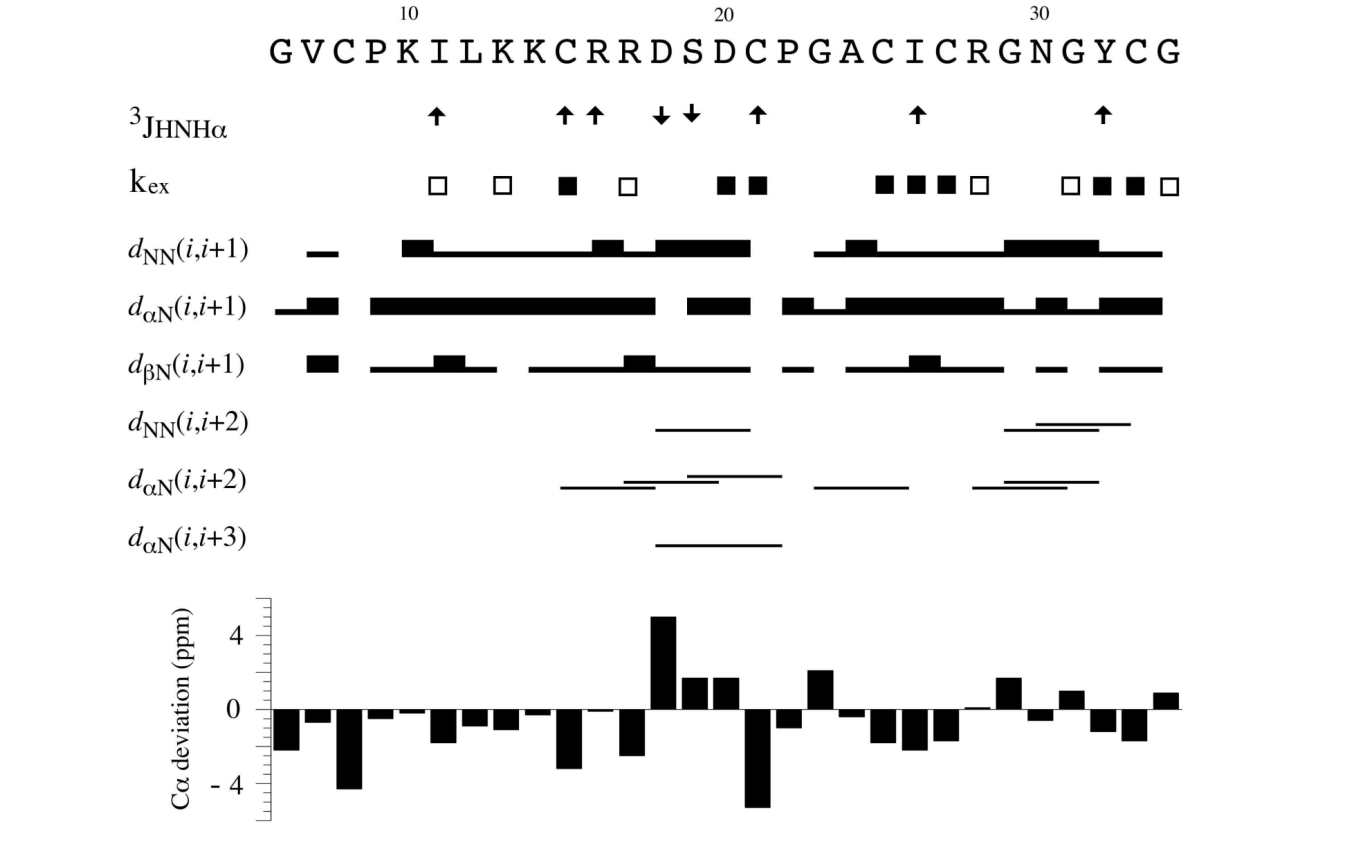
**NMR data summary for lin-McoTI**. Data on sequential and medium range NOE connectivities, ^3^J_HN-H*α *_coupling constants and slowly exchanging amide protons observed for lin-MCoTI are summarized. The height of the bars correspond to the strength of the NOEs. The values of the ^3^J_HN-H*α *_coupling constants are indicated by ↓ (< 4 Hz) and ↑ (> 8.5 Hz). Open and filled squares indicate backbone amide protons that were still observed after 3 and 24 h, respectively, in ^2^H_2_O. The deviations from random coil values for the ^13^C chemical shifts of C*α *of lin-MCoTI are plotted at the bottom of the figure.

The assignment of all the ^1^H and ^13^C resonances present in the spectra was achieved using well-established techniques [31] and part of the ^1^H sequential assignment for lin-MCoTI is provided as additional file 1: NMR spectra of lin-MCoTI. ^1^H and ^13^C chemical shifts are available from additional file 2: Chemical shifts in ppm for lin-MCoTI. Figure 2 summarizes the sequential and medium range nuclear Overhauser effects (NOEs), ^3^J_HN-H_*α *coupling constants, slowly exchanging amide protons and the C*α *chemical shift differences from random coil values for the lin-MCoTI peptide [32,33]. The observation of two small ^3^J_HN-H_*α *coupling constants for residues Asp^18 ^and Ser^19^, and the d_NN_(i, i+2), d_*α*N_(i, i+2) and d_*α*N_(i, i+3) NOEs in the region 16–21 shows the presence of a short 3_10 _helix which is generally detected between the second and the third cysteine of squash inhibitors. The nuclear magnetic resonance (NMR) parameters measured in the region 22–25 are in agreement with a *β*-turn (d_*α*N_(i, i+2) NOE and slowly exchanging amide proton of residue 25). It is now well-established that squash inhibitors share a common structural motif with other knottins. This Cystine Stabilized *β*-sheet (CSB) motif is essentially made of an anti-parallel triple-stranded *β*-sheet [1,34]. The CSB motif is well-defined in EETI-II and lin-MCoTI and is similar to the corresponding region in cyclic MCoTI-II [26]. The three regions of the sequence involved in this motif are 13–15, 26–28 and 32–34. Large ^3^J_HN-H*α *_coupling constants and slowly exchanging amide protons measured in these parts of the sequence are in agreement with this structure. In addition, all the characteristic inter-strand NOEs were detected. Region 26–34 is a *β*-hairpin with a *β*-turn involving residues 28–31 that was ascertained by the presence of d_NN_(i, i+2) and d_*α*N_(i, i+2) NOEs.

**Figure 3:**
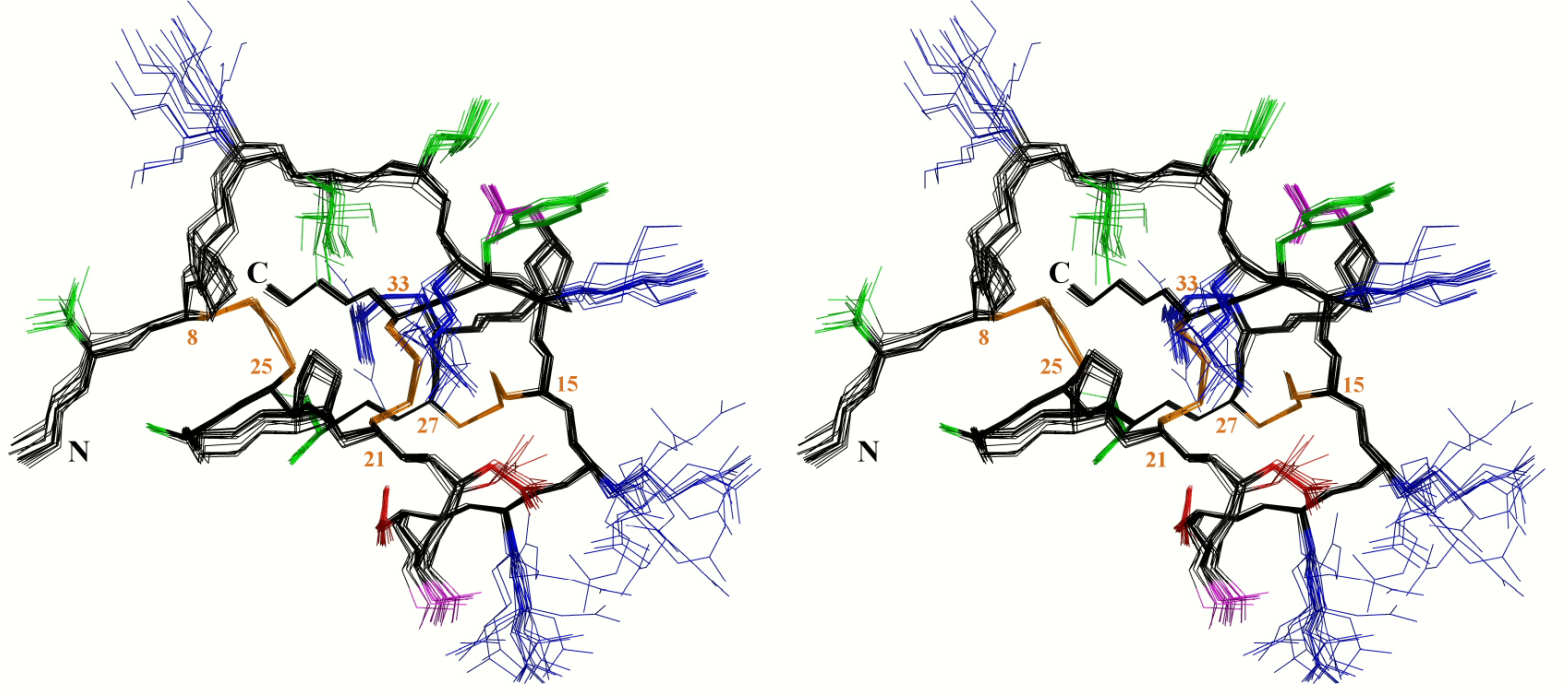
**Stereoview of the 20 lowest energy solution structures of lin-McoTI**. Structures have been superimposed for their C*α *atoms. The coloring scheme is as follows: whole backbone and proline, black; hydrophobic and aromatic residues, green; polar residues, magenta; acidic residues, red, basic residues, blue; disulfide bridges, orange. Cysteines and N- and C-termini are labeled.

The three-dimensional structures of lin-MCoTI and EETI-II were determined from NMR data using the same strategy previously used for structural studies of the cyclic wild type MCoTI-II [26]. The NMR study led to 78 and 71 sequential and 167 and 122 medium and long range NOEs for lin-MCoTI and EETI-II, respectively. Eight (lin-MCoTI) and thirteen (EETI-II) *φ *angles were determined from the ^3^J_HN-H*α *_coupling constants, and stereospecific assignment of the H*β *protons was achieved for 9 residues (Figure 2 and Table 1). The connectivity of the disulfide bonds for lin-MCoTI was deduced on the basis of the very strong sequence similarity with the parent compound. It is also strongly supported by the similarity between the NMR data sets for MCoTI-II and lin-MCoTI. Moreover, no alternative disulfide connectivity has yet been reported for miniproteins with the knottin fold [35-37]. The NMR data were converted into distance and angle constraints and the three-dimensional (3D) structures were obtained as described in Methods. The 30 lowest energy (molecular mechanics plus restraint energy) structures were selected to represent the NMR structures of lin-MCoTI and of EETI-II. The statistics for constraint violations and for molecular mechanics energies are shown in Table 1. Twenty lin-MCoTI solution structures are displayed in Figure 3.

**Table 1:** Statistics on geometry, energy and NMR data of the EETI-II and lin-MCoTI solution structures

	**lin-MCoTI^a^**	**EETI-II^a^**
**Experimental constraints**		

Distances^b^		
short	78	71
medium & long range	167	122
Dihedrals^c^		
Phi -90/-40°	17, 18	17, 18, *22*
Phi -160/-80°	10, 14, 15, 20, 25, 31	10, *12*, 14, 15, 20, *24*, 25, *26*, *29*, 31
Chi1 120/270°	7, 26	7,26
Chi1 -120/0°	17, 20, 24, 31, 32	17, *19*, 20, 24, 31, 32
Chi1 0/120°	*14*,29	29

**Constraint violations^d^**		

Distances		
number > 0.2 Å	0	0
number < 0.2 Å	6.8 (0.7)	2.9 (0.9)
maximum (Å)	0.17 (0.02)	0.15 (0.02)
Dihedral		
number > 2°	0	0

**AMBER energies (kcal mol^-1^)**		

Bond	15.15 (0.24)	13.51 (0.33)
Angle	42.8 (1.3)	39.90 (1.48)
Dihedral	238.9 (1.2)	224.2 (2.3)
van der Waals	-177 (1.8)	-163.9 (2.2)
Electrostatic	-1494.5 (17)	-1738.5 (34)
Generalized Born	-727.2 (14.3)	-446.0 (30.8)
Surface based	10.7 (0.2)	10.7 (0.3)
Total AMBER	-827.3 (2.5)	-799.6 (2.0)
Constraint	3.08 (0.29)	1.67 (0.23)

**PROCHECK statistics**		

Residues in most favored regions (A, B, L)	91.1%	95.1%
Residues in additional allowed regions (a, b, l, p)	8.9%	4.9%

**Deviations from ideal geometry**		

Bond	0.010 (10^-4^)	0.010 (10^-4^)
Angle	1.88 (0.032)	1.89 (0.040)

^a^Values in parentheses indicate standard deviations. ^b^Number of constraints. ^c^Residue numbers. Residues in italic correspond to constraints that are not present in both compounds. ^d^Average distance violations in Å.

The calculated structures of lin-MCoTI and of EETI-II satisfy the NMR data very well with no distance and dihedral violation larger than 0.2 Å or 5°, respectively (Table 1). Statistical analyses using the PROCHECK-NMR software [38] show that the overall stereochemistry of the lin-MCoTI and EETI-II solution structures is correct with all non-glycine and non-proline residues lying in the most favored and additional allowed regions of the Ramachandran map (Table 1). As expected, the refined models also display large negative molecular mechanics energies.

The lin-MCoTI and EETI-II solution structures are well-resolved with pairwise RMS deviations of 0.26 Å and 0.34 Å for superimposition of backbone atoms of residues 8–33, respectively (Table 2). The two average solution structures are close to the EETI-II X-ray structure (PDB ID: 1w7z[39]) with RMS deviations of 0.68 and 0.74 Å for backbone atoms of residues 8–33, i.e. all residues except the C-to-N linker (Table 3). The structures are also close to the cyclic MCoTI-II structure with RMS deviations of 0.78 Å and 0.87 Å, respectively. The EETI-II, lin-MCoTI and MCoTI-II solution structures are available from the Protein Data Bank [28] under PDB IDs 2it7, 2it8, and 1ha9, respectively. The refined EETI-II solution structure described here (PDB ID 2it7) significantly differs from the distance geometry structure published in 1989 (PDB ID 2eti) with RMS deviations of 1.9 Å for backbone atoms of residues 8–33. The main difference lies in the inhibition loop (residues 8–14) as shown by the much lower RMS deviation of 1.0 Å obtained when the loop is omitted from the superimposition.

**Table 2:** Structural variations of backbone atoms (N, C*α*, C, O) in NMR and MD conformational ensembles

	**Lin-MCoTI**		**MCoTI-II**			**EETI-II**	
**Residues**	**8–33**	**15–33**	**1–34**	**8–33**	**15–33**	**8–33**	**15–33**
**NMR^a, b^**	0.26 (0.12)	0.15 (0.06)	1.18 (0.36)	0.51 (0.16)	0.28 (0.09)	0.34 (0.13)	0.13 (0.06)
**MD^c^**							
300 K	0.61	0.48	0.91	0.68	0.46	0.53	0.40
400 K	0.97	0.64	1.26	0.80	0.58	1.10	0.64
500 K	2.04	1.61	1.97	1.25	0.82	1.67	1.33

^a^Average pairwise RMS deviations in Å. ^b^Values in parentheses indicate standard deviations. ^c^Average atomic positional fluctuations in Å.

**Table 3:** RMS deviations between average structures

		**NMR**			**MD**			**X-ray**
		**MCoTI-II**	**lin-MCoTI**	**EETI-II**	**MCoTI-II**	**lin-MCoTI**	**EETI-II**	**EETI-II**
**NMR**	**MCoTI-II**	-	0.52	0.69	0.65	0.61	0.85	0.62
	**lin-MCoTI**	0.78	-	0.62	0.81	0.70	0.77	0.61
	**EETI-II**	0.87	0.69	-	0.93	0.87	0.81	0.54
		
**MD**	**MCoTI-II**	1.02	0.95	1.15	-	0.67	0.84	0.71
	**lin-MCoTI**	0.95	0.79	1.06	0.80	-	0.48	0.64
	**EETI-II**	1.14	0.83	1.08	0.89	0.59	-	0.52
		
**X-ray**	**EETI-II**	0.90	0.68	0.74	0.72	0.71	0.61	-

Values in Å for superimposition of backbone atoms of residues 15–33 (above diagonal) and 8–33 (below diagonal).

### Molecular dynamics simulations of MCoTI-II, lin-MCoTI and EETI-II

**Figure 4:**
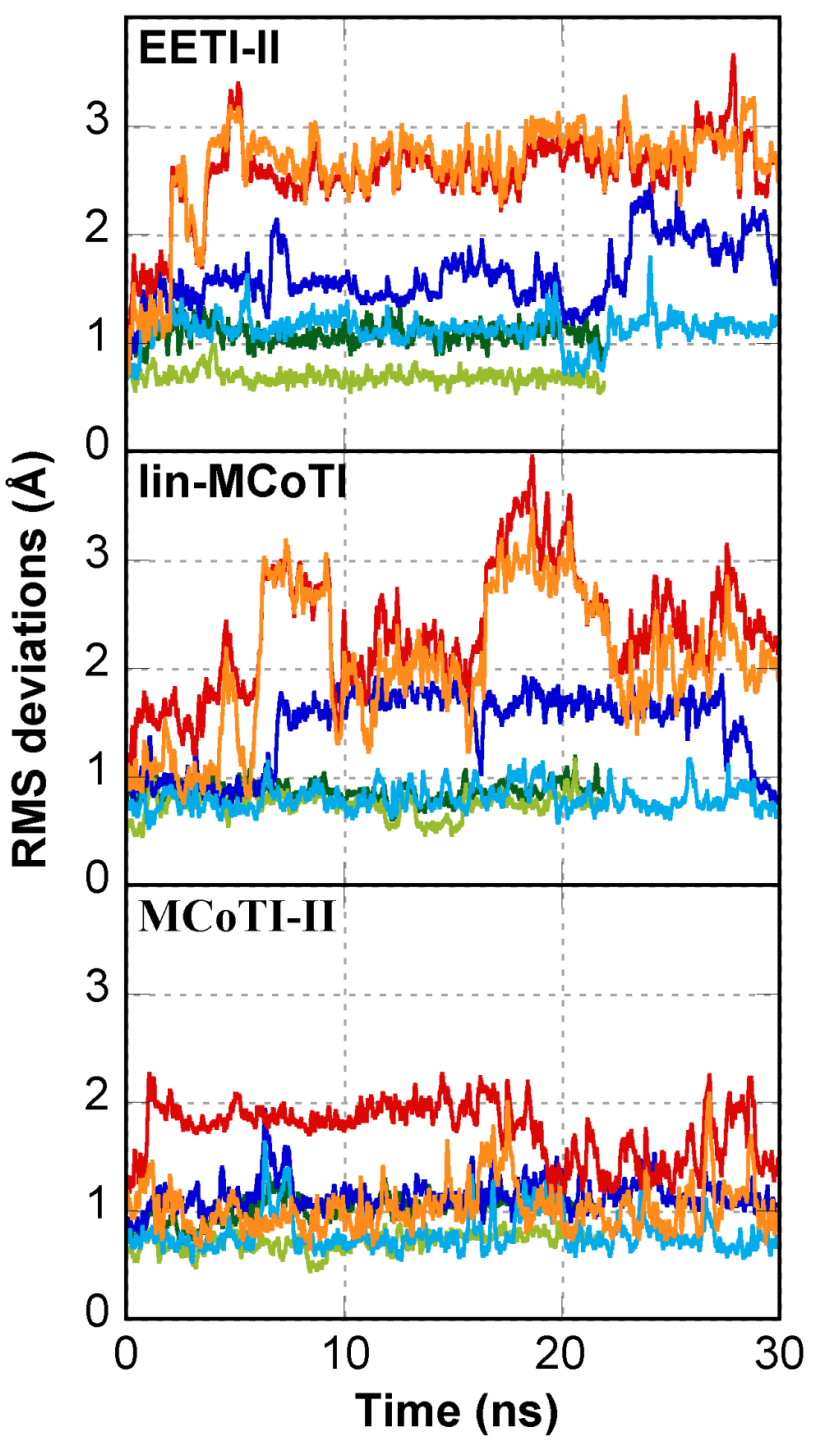
**Root mean square deviation from the NMR conformation along the MD simulations**. Reported values are for backbone atoms (N, C*α*, C, O) of residues 8 to 33 at 300 K (green), 400 K (blue) and 500 K (red). Conformations were superimposed for residue ranges 8–33 (heavy colors) and 15–33 (light colors).

**Figure 5:**
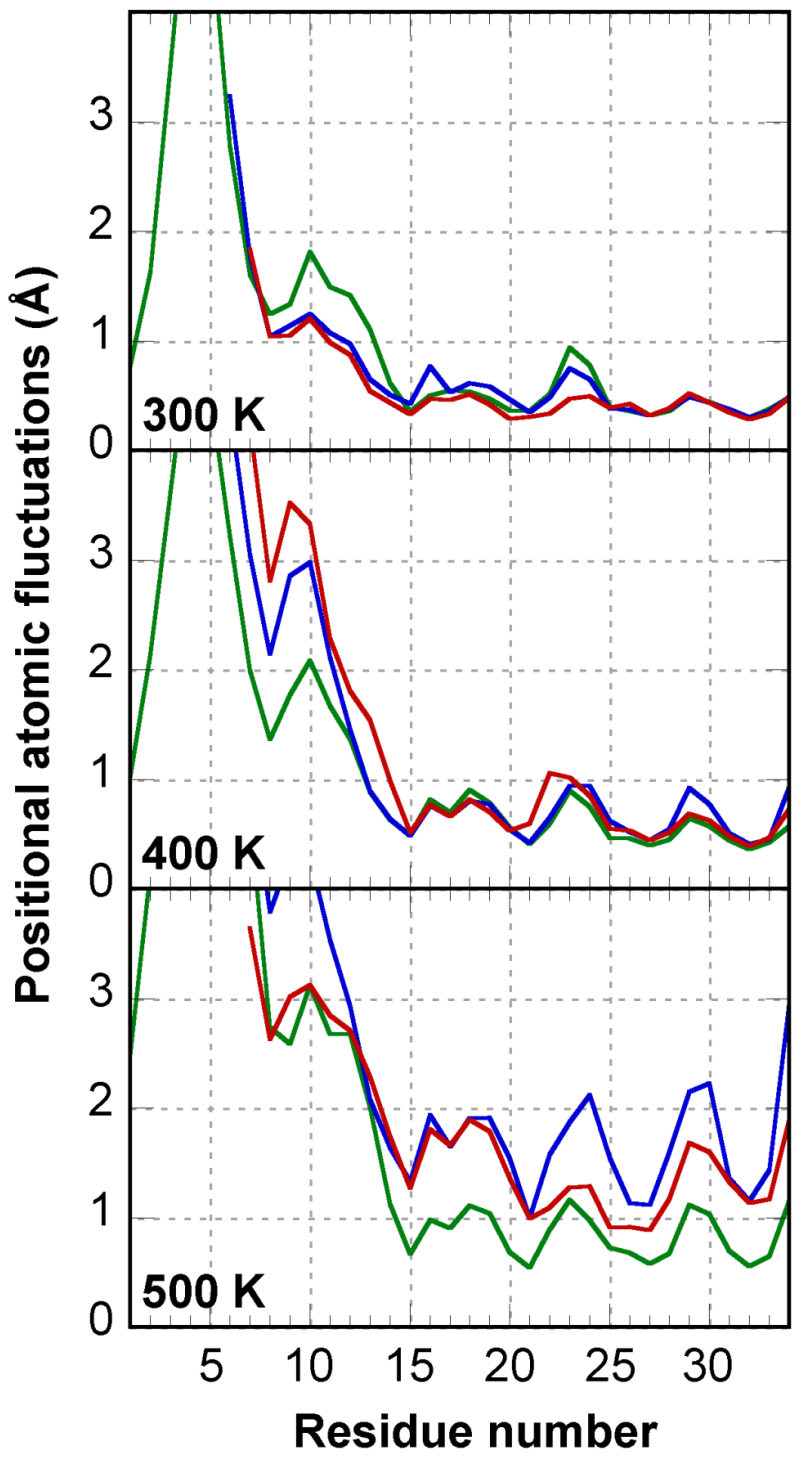
**Root mean square positional atomic fluctuations in the 300 K, 400 K and 500 K MD simulations**. Reported values are for backbone atoms (N, C*α*, C, O) and per residue: MCoTI-II (green line), lin-MCoTI (blue line), EETI-II (red line).

**Figure 6:**
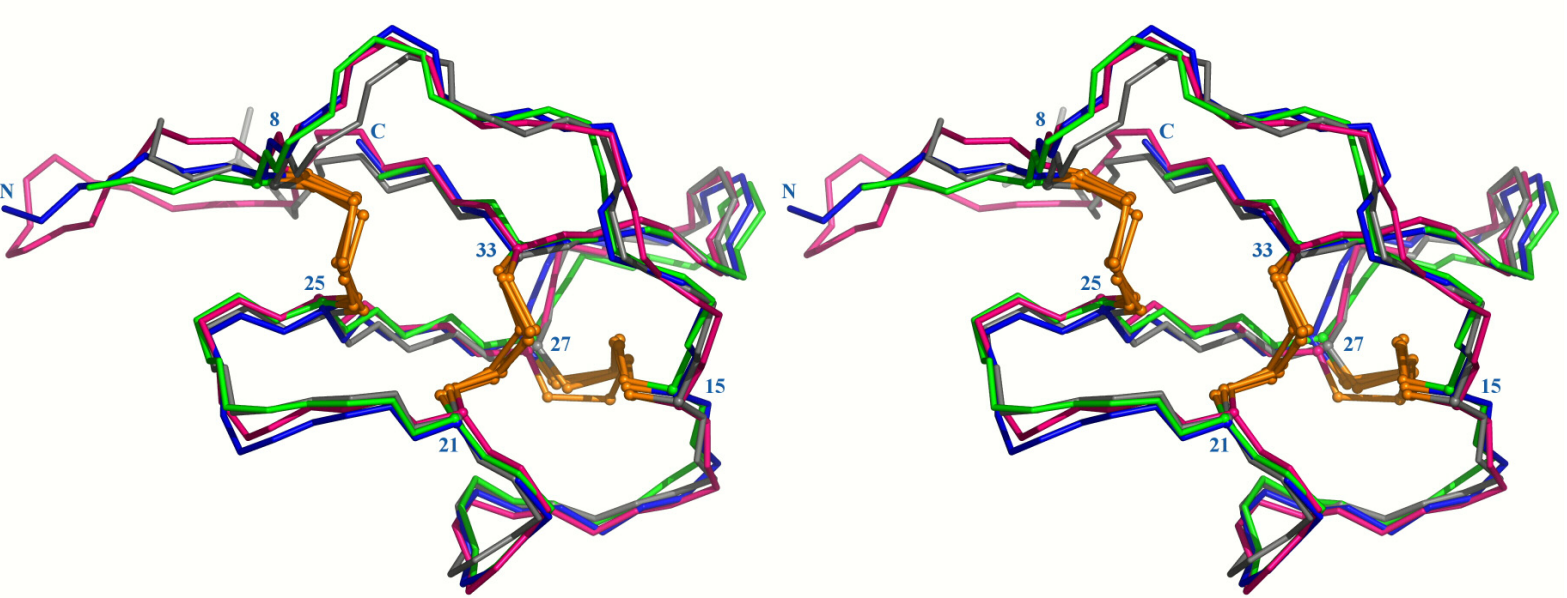
**Stereoview of average structures from the 300 K simulations**. Structures were superimposed on top of the EETI-II X-ray structure (PDB ID: 1w7z[39]), shown in grey, for backbone atoms of residues 15–33. EETI-II is shown in green, MCoTI-II in red, and lin-MCoTI in blue. Cysteines and N- and C-termini of lin-MCoTI are labeled. Disulfide bridges are shown as orange ball-and-stick representations.

Rather than comparison of the structures, one major goal of our study was to determine to which extent the miniprotein flexibility is affected by the circularization. Therefore, the lowest energy solution structures of MCoTI-II, lin-MCoTI and EETI-II were submitted to 22ns-long unrestrained molecular dynamics (MD) simulations in periodic boxes of explicit water molecules at 300 K using the program AMBER [40]. RMS deviations and positional atomic fluctuations were computed on all saved conformations after structural superimposition and are detailed in Table 2, Figure 4 (green lines), and Figure 5 (top). For each simulation, the conformation closest to the average structure was used for comparison with other structures (Table 3 and Figure 6).

The average solution structures are slightly closer to the X-ray EETI-II structure than the MD average structures (Table 3). The fact that no experimental restraints were applied during the dynamics might explain part of the difference. The EETI-II NMR structure is closer from the X-ray structure than the MCoTI-II and lin-MCoTI structures for the 15–33 region (i.e. the CSB motif), but not for the 8–33 region with RMS deviations of 0.54 Å and 0.74 Å, respectively (Table 3). The RMS deviations are displayed in Figure 4, green lines. All three compounds display low deviations from the initial conformation, close to 1 Å. Interestingly, the linear EETI-II showed the lowest deviation for the CSB motif (residues 15–33).

To evaluate the flexibility of each compound, the per-residue atomic fluctuations in the three simulations have been plotted and are displayed in Figure 5 (top). The most striking feature is that the head-to-tail linker in cyclic MCoTI-II is by far the most flexible region. Clearly, the cyclization does not modify the flexibility significantly. This observation is consistent with previous NMR structure calculations [25,26]. Except for the linker, the three compounds display similar flexibilities. But, interestingly, data in Table 2 and Figure 5 (top) show that the two linear peptides display the lowest average fluctuations although differences remain limited. Lin-MCoTI appears slightly more mobile than EETI-II in the 8–21 region corresponding to the inhibitory loop and the 3_10 _helical loop. The fact that the linear EETI-II is the less flexible compound strongly suggests that head-to-tail cyclization is not a key element of structure stabilization in these conditions. Hydrogen bonding in the simulations are summarized in Table 4. Analysis of hydrogen bonding reinforces previous analyses since the percent occurrences and the average donor-acceptor distances are not significantly affected by the cyclization.

**Table 4:** Hydrogen bond occurrences during the molecular dynamics simulations

**Local structure**	**Residues**	**MCoTI-II**		**lin-MCoTI**		**EETI-II**	
**3-stranded *β*-sheet**	N33-O13	68.7	3.03	97.6	2.98	**99.7**	2.95
	N15-O31	98.7	2.94	**98.9**	2.93	98.8	2.97
	N26-O34	99.9	2.88	99.9	2.90	**100.0**	2.74
	N34-O26	99.5	2.91	**99.6**	2.89	97.2	2.98
	N28-O32	**95.9**	2.98	91.7	3.02	93.5	3.01

***β*-turns**	N25-O22	88.8	3.06	**92.3**	3.05	89.6	3.13
	N25-O23	7.5	3.20	5.1	3.21	**21.6**	3.22
	N31-O28	**97.0**	3.03	96.4	3.04	96.7	3.06

**3-10 helix**	N20-O17	83.8	3.15	87.2	3.13	**97.7**	3.05
	N21-O18	**81.9**	3.17	67.8	3.19	70.6	3.19
	N21-O19	-	-	**5.6**	3.15	0.2	3.35

**C to N linker**	N6-O3	**36.5**	3.15	-	-	-	-
	N6-O4	**15.7**	3.18	-	-	-	-

**Others**	N10-08	**8.6**	2.99				
	N11-O9	51.2	2.93	**73.4**	2.94	29.2	3.03
	N13-O11	13.5	3.12	**22.1**	3.19	9.2	3.06

**Side chains**	N16-Asp^20^	76.1	2.90	81.6	2.94	**98.7**	2.88
	N17-Asp^20^	71.7	3.01	50.5	3.02	**88.3**	3.04
	N27-Asp^18^	**93.7**	2.90	90.9	2.92	22.0	2.97
	Arg^16^-Asp^20^	-	-	**21.5**	3.00	5.8	2.99
	Arg^17^-Asp^20^	**23.2**	2.97	5.3	2.95	(Gln^17^)	
	Lys^13^-Asp^20^	**10.1**	2.96	-	-	(Met^13^)	
	N32-Asn^30^	68.8	3.16	70.1	3.16	**79.6**	3.15
	Lys^13^-O20	32.1	2.89	**44.2**	2.89	(Met^13^)	
	Lys^13^-O14	15.8	2.93	**30.8**	2.94	(Met^13^)	
	Lys^14^-O30	-	-	-	-	**7.3**	3.02
	Lys^14^-O31	-	-	-	-	**7.4**	3.09
	Lys^10^-O8	-	-	-	-	**6.6**	2.96
	Arg^16^-O15	**10.3**	2.95	-	-	-	-
	Arg^28^-O1	**50.5**	3.01	-	-	(Gly^28^)	
	Arg^28^-O32			**22.7**	3.14	(Gly^28^)	
	Arg^28^-O33	12.2	3.03	**55.9**	3.05	(Gly^28^)	

For each hydrogen bond, the percentage of occurrence is followed by the average distance between heavy atoms (in Å). Only hydrogen bonds that occur more than 5% of the time are reported using 3.5 Å and 120° as distance and angle cut-offs, respectively. Bold numbers indicate the highest percentages in each row. Nx and Ox refer to the amide and carbonyl groups of residue x, respectively. When side-chains are involved, the residue is indicated using the three letter code.

### Experimental and simulated thermal unfolding

**Figure 7:**
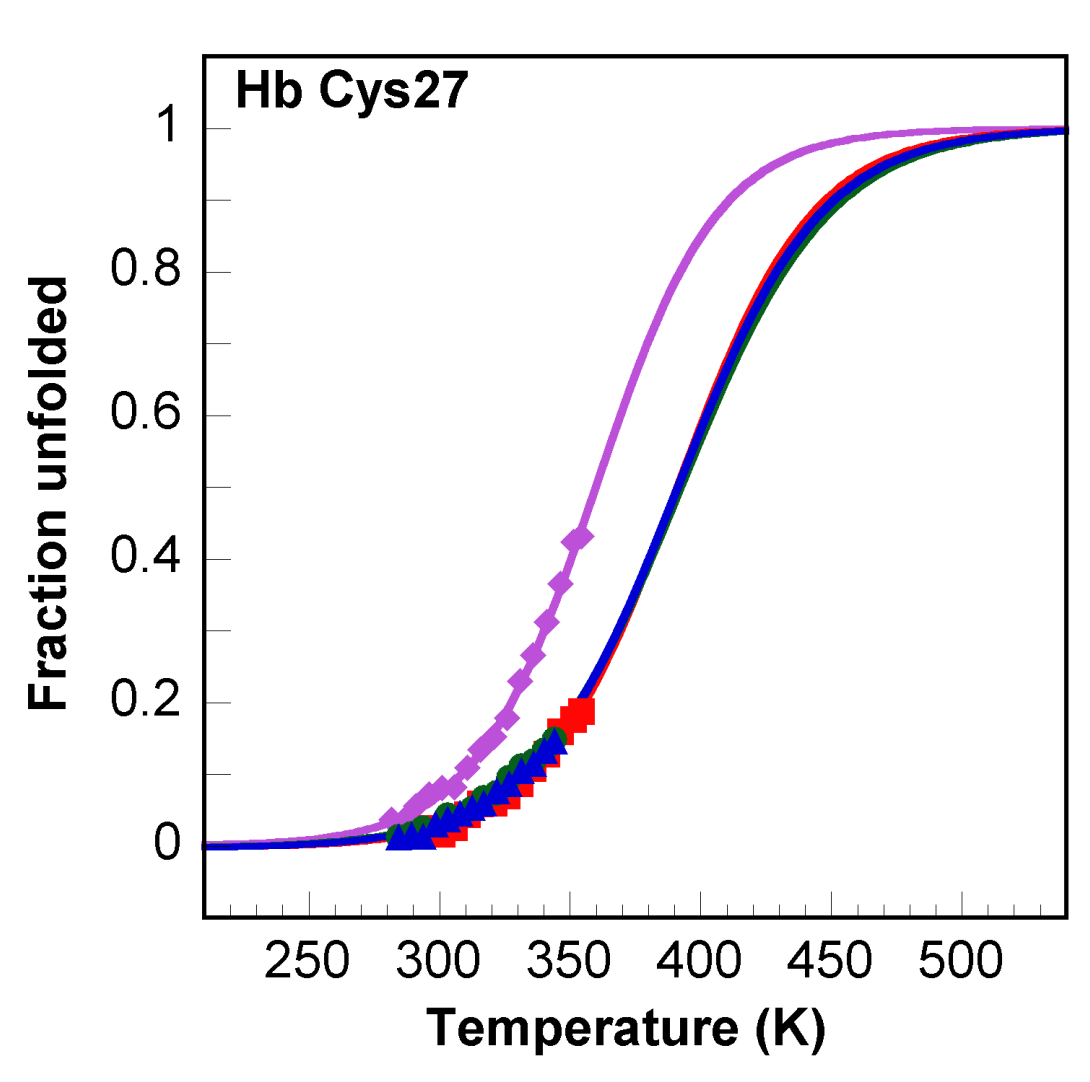
**Thermal unfolding curves**. The fraction unfolded calculated from the chemical shift (see Methods) is plotted as a function of temperature. Protein identification is as follows: Min-23 (purple, ◆), EETI-II (red, ■), MCoTI-II (green, ●), lin-MCoTI (blue, ▲).

NMR thermal unfolding experiments were performed on wild type circular MCoTI-II and on the linear analog. In these analyses, the chemical shift variations due to temperature increases between 10°C and 80°C are considered to result solely from a simple two-state unfolding mechanism. It is worth noting that the thermal unfolding has been studied without reduction of disulfide bridges since reduced squash inhibitors are essentially unstructured [16]. The chemical shift for the theoretical fully unfolded species is approximated as the random coil values for short peptides. Protons that could be followed in accumulated spectra at various temperatures were used to compute Tm values [34,41]. Unfolding curves calculated for the H*β *proton of Cys^27 ^in cyclic MCoTI-II and its linear analog are displayed in Figure 7, and Tm values calculated from data recorded for few protons are tabulated in Table 5. Data from previous experiments on linear EETI-II and on a shorter analog Min-23 [34], a derived two-disulfide peptide containing the CSB motif (Figure 1), have been included for comparison. As expected, the shorter two-disulfide Min-23 peptide consistently displays lower Tm values by approximately 30°C [34]. On the other hand, the differences between the three other compounds appear to be marginal and to fall within experimental errors, thus precluding any detailed comparisons. Although no large stability increase due to cyclization is apparent from unfolding experiments no definitive conclusion could be drawn.

**Table 5:** Estimated Tm values from NMR thermal unfolding

**Proton**	**MCoTI-II**	**lin-MCoTI**	**EETI-II**	**Min-23**
**H*β *Cys27**	133 (4)	130 (4)	127 (3)	91 (1)
**H*α *Gly31**	127 (5)	124 (9)	-	111/117 (2)
				
**H_2,6 _Phe/Tyr32**	133 (8)	118 (21)	153 (26)	97 (1)
**H_3,4,5 _Phe/Tyr32**	115 (5)	118 (7)	-	113/86 (3)
				
**H*α *Cys33**	152 (8)	144 (8)	-	102 (10)

Mean Tm values in °C, obtained from hundred calculations using randomly picked chemical shifts within a -0.02/+0.02 range around the experimental values, are reported. Standard deviations are shown within parentheses.

**Figure 8:**
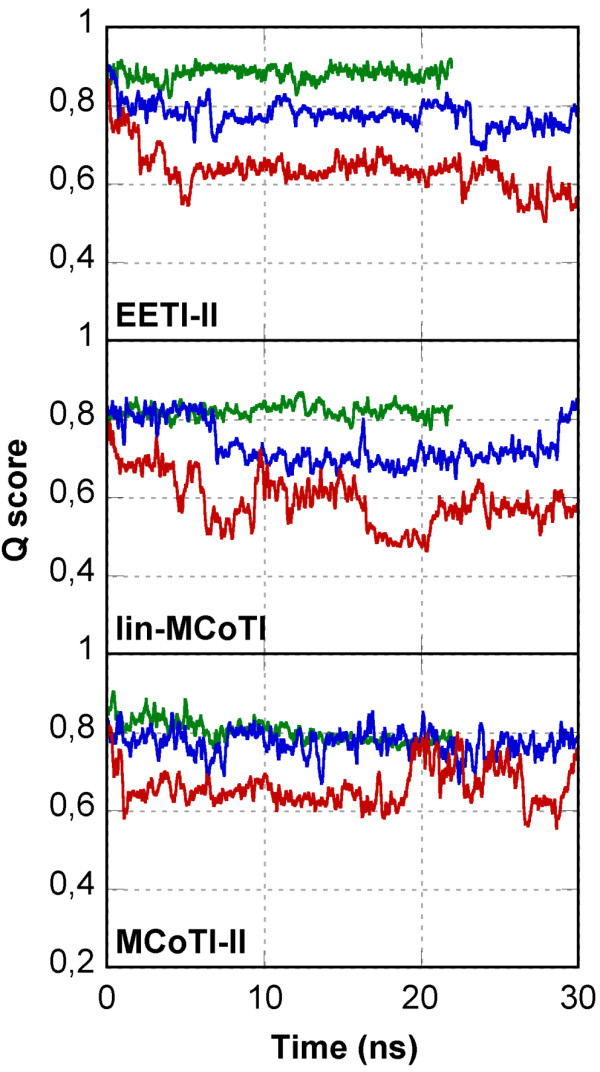
**Q-scores of conformations explored in the unfolding simulations**. The minimized starting NMR conformation is used as the reference native structure. For each compound, the Q-score evolution is shown at 300 K (green), 400 K (blue) and 500 K (red).

**Figure 9:**
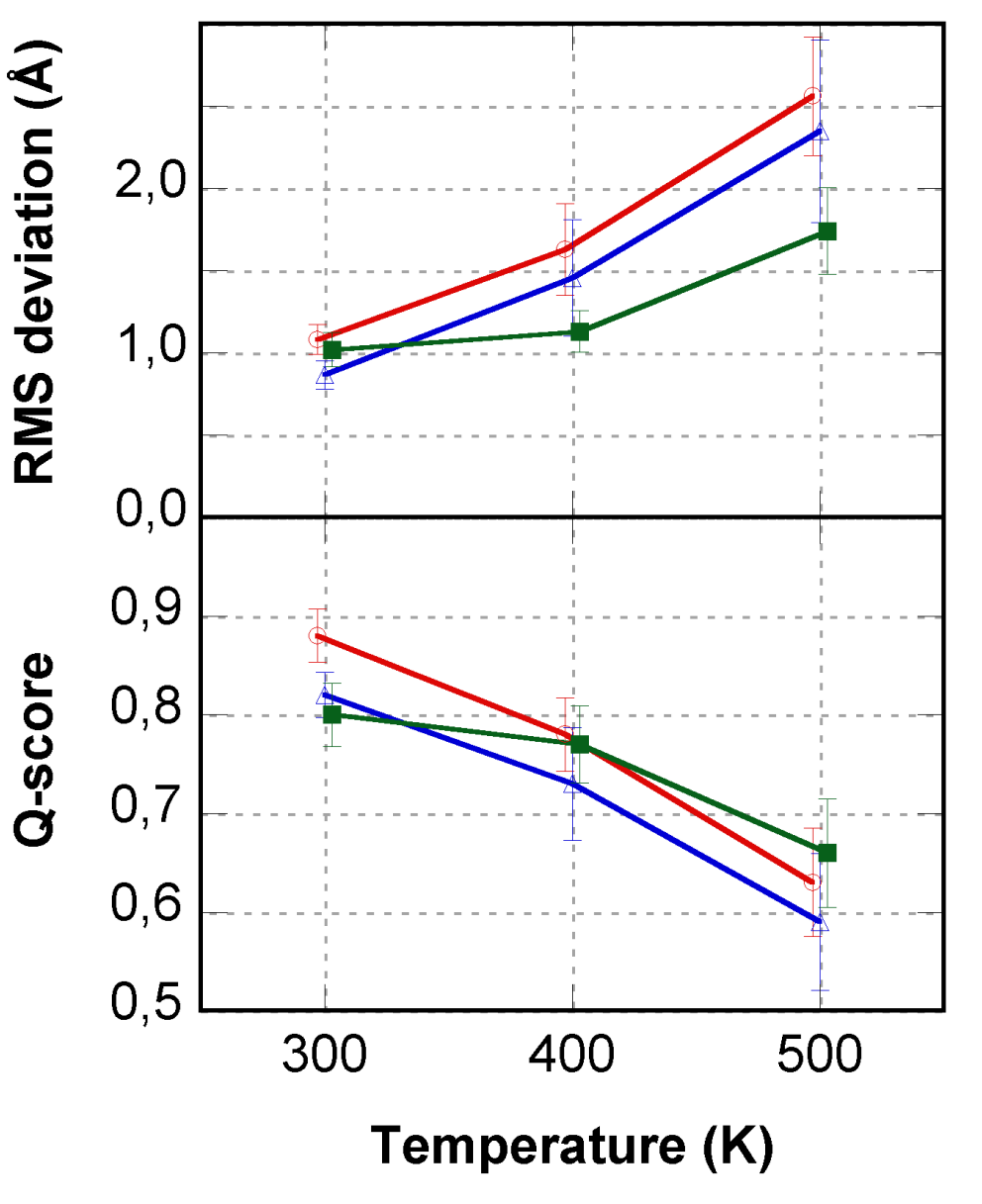
**Variation of the mean Root Mean Square deviations and Q-scores with temperature**. MCoTI-II is shown as green lines, lin-MCoTI as blue lines and EETI-II as red lines.

To provide further information, unfolding simulations were performed using molecular dynamics simulation at high temperature (400 K and 500 K). The RMSDs from the starting conformations are shown in Figure 4 (blue and orange-red curves), and a nativeness score, the Q-score [42], in Figure 8. The Q-score has been used as a measure of structural similarity to the native conformation and indicates the fraction of native non-bonded contacts. In the Q-score implementation used here, C*α *inter-residue distances are used as contact measures. The use of other implementations based on all heavy atom distances led to very similar results (data not shown). A Q-score equal to 1 means that the structure is fully native whereas a Q-score below 0.4–0.6 means that the structure is significantly unfolded or incorrectly folded. Average RMSDs and Q-scores plotted as a function of temperature are displayed in Figure 9. The RMSD from the starting structure and the nativeness Q-score criterion are not independent and display similar profiles. While the two linear knottins display close results with almost identical slopes for variation of RMSD or Q-score with temperature, the cyclic MCoTI-II appears less sensitive to temperature increase (Figure 9). It is likely that the constraints imposed by the covalent connection between the N- and C-termini, in addition to the disulfide bridges, slow down the unfolding process during the highest temperature (500 K) simulation. Comparison of the RMSDs of the Cystine-Stabilized Beta-sheet motif in the simulations at 500 K (orange curves in Figure 4) highlights drastic differences between the linear and the cyclic compounds. Despite the constraints imposed by the conserved disulfide bridges, significant unfolding of the CSB motif (residues 15–33) is apparent for the linear compounds with average RMSD values between 2 and 3 Å. In contrast, the CSB motif of the cyclic compound is essentially conserved in the simulation at 500 K with RMSD values that remain as low as 1 Å.

## Discussion

The 3D structure of squash inhibitors is known since the late 1980s [29,30,43]. They were soon recognized as a prototypic member of a structural class of disulfide-rich miniproteins known as the knottins or Inhibitor Cystine Knots (ICK) [44,45]. The first cyclic knottin, kalata B1, was described shortly after, in which the N- and C-termini are connected through a regular peptide bond, yielding a head-to-tail macrocyclic and knotted peptide. Although this circularization could have been an exception, it soon appeared that macrocyclic knottins were common in plants in the *Rubiaceae *and *Violaceae *families, constituting the large cyclotide family [10,11,46]. The only other example of macrocyclic knottin has been found more recently in the squash inhibitor family which otherwise contains more than thirty linear miniproteins [1,3,13]. Until now, the structural and functional impact of the circularization remains poorly understood, and very little is known on dynamics and thermal stability of cyclic squash inhibitors when compared to their linear counterparts. This prompted us to investigate structures, dynamics and stabilities of cyclic and linear squash inhibitors and analogs.

### NMR experiments and molecular dynamics simulations evidence a limited impact of cyclization on structure and dynamics

The average NMR structures of the three studied compounds are close from each other with most RMS deviations lying below 0.9 Å for backbone atoms of residues 8–33 (Table 3). The differences between NMR and MD structures are only slightly higher and mostly remain near 1.0 Å, a reasonably low value. Moreover, all NMR and MD structures are close to the EETI-II X-ray structure with RMS deviations below 0.9 Å. MD simulations are expected to provide a more energetically rigorous conformational sampling than NMR structure determination. This is mainly because NOE averaging on the NMR time scale emphasizes short distances and because instantaneous and continuous application of these average constraints biases the conformational sampling towards shorter distances. Since no unrealistic deviation occurred during the simulations, the MD conformations were used for comparisons and assessment of flexibilities.

A superimposition of the average MD structures from simulations at 300 K onto the EETI-II X-ray structure is displayed in Figure 6. The only significant difference between EETI-II and MCoTI-II, beside the cyclization, concerns the 22–25 turn. A PROMOTIF [47] analysis indicates that this *β*-turn is of type I in EETI-II but of type II' in MCoTI-II and lin-MCoTI. This modification is likely a consequence of the different turn sequences (LAGC and PGAC in EETI-II and MCoTI-II, respectively). Interestingly, despite their flexibility, the inhibitory loops of EETI-II, MCoTI-II and lin-MCoTI (residues 8–15) remain reasonably close to the conformation in the EETI-II X-ray structure (Figure 6).

In all three compounds, the Asp^20 ^side chain forms strong hydrogen bonds with amides of residues 16 and 17, but, surprisingly, the percent occurrences are lower in MCoTI-II and lin-MCoTI (Table 4). It is worth noting, however, that in MCoTI-II and lin-MCoTI, Asp^20 ^is surrounded by four positively charged residues at positions Lys^13^, Lys^14^, Arg^16 ^and Arg^17^. Instead of Lys^13 ^and Arg^17^, corresponding residues in EETI-II are Met and Gln, respectively. Examination of Asp^20 ^interactions in MCoTI-II and lin-MCoTI (Table 4) shows that, beside interactions with backbone amides of residues 16 and 17, Asp^20 ^is also interacting with the Arg^17 ^side chain in both compounds (percent occurrences 23.2 and 5.3, respectively), and with Arg^16 ^or Lys^13 ^in lin-MCoTI and MCoTI-II, respectively (percent occurrence 21.5 and 10.1). The latter interaction, which necessitates a small displacement of the Asp^20 ^carboxylate, supports a higher flexibility of lin-MCoTI in this region, in agreement with the residue fluctuations shown in Figure 5. In contrast to the stronger Asp^20^-amides interactions in EETI-II, the hydrogen bonding of the Asp^18 ^side chain with the amide of residue 27 is much weaker in EETI-II (22%) than in the two other compounds (94 and 91% in MCoTI-II and lin-MCoTI, respectively). This might be related to the H-bonding in the 3–10 helix, with both stronger (N20-O17, 97.7%) and weaker (N21-018, 70.6%) interactions in EETI-II.

In general, there are no large differences in hydrogen bonding (percent occupancy and average distance) between MCoTI-II and lin-MCoTI. However, in MCoTI-II, the Arg^28 ^side chain is hydrogen bonding 50% of the time with the carbonyl of Ser^1 ^(Table 4), a residue which does not exist in lin-MCoTI. Instead, in lin-MCoTI, Arg^28 ^is hydrogen bonding with the carbonyl of Cys^33 ^and, to a minor extent, Tyr^32 ^(Table 4). There is no such interaction in EETI-II since residue 28 is a glycine. The 3_10 _helix region also differ slightly with weaker N21-O18 and N17-Asp^20 ^hydrogen bonds in the linear compound (Table 4). This difference is surprising because MCoTI-II and lin-MCoTI sequences are strictly identical on this side of the molecule. It thus appears that the modifications near the N- and C-termini propagate toward the 3_10 _helix, probably via subtle modifications of the hydrogen bonding scheme due to modified charges and hydrogen bond acceptors near the C-terminus. This observation is consistent with the fact that the amide proton of residue Arg^17 ^is exchanged more rapidly in lin-MCoTI than in MCoTI-II. In fact several other amide protons display a similar behavior, in particular those of Arg^28^, Gly^31 ^and Gly^34^, that all belong to the triple-stranded *β*-sheet (Figure 2 and [26]), indicating a slightly higher flexibility of the sheet in lin-MCoTI.

### Cyclic and linear squash inhibitors exhibit close thermal stabilities

With the hope to better evaluate the influence of cyclization on stability, we have performed NMR-monitored thermal unfolding experiments on MCoTI-II and lin-MCoTI. Due to the extremely high stabilities of the compounds, only limited unfolding could be achieved at temperatures compatible with NMR experiments which resulted in large uncertainties in curve fitting and calculation of thermodynamics parameters. From results in Table 5, it appears that, as expected [34], the shorter two-disulfide Min-23 peptide is clearly less stable than the three-disulfide compounds with Tm values lowered by about 10–40°C. In contrast, differences between Tm values for the three-disulfide knottins are smaller than standard deviations and may not be significant. Nevertheless, most calculated Tm values for the circular MCoTI-II are slightly higher than those calculated for the linear analog, suggesting a small stability increase in the circular compound. This would be consistent with the results of the MD simulations that showed a small flexibility increase in lin-MCoTI, and with the NMR data indicating a faster exchange of several amide protons in this compound.

However, (i) even the highest temperature used for NMR unfolding experiments remained too low to achieve a significant overall unfolding, (ii) the thermodynamics data are scarce and, (iii) more importantly, the two-state unfolding hypothesis and the use of the random coil values for chemical shifts of the unfolded species may not be valid in this case, especially because of the high disulfide bridge content. It is worth noting also that, for the cyclotide kalata B1, no significant changes were observed in circular dichroism spectra in absence or presence of 8 M urea and in the temperature range 5–90°C [24]. Therefore, to get further information on stability while avoiding the experimental limitations, we have used high temperature molecular dynamics simulations that make possible to study processes that are difficult to investigate experimentally.

### Cyclic MCoTI-II displays better thermoresistance to unfolding

It has been suggested that molecular dynamics simulations at high temperature accelerate protein unfolding without changing significantly the unfolding pathway and provide useful information on thermal events [48-51]. Therefore, in addition to the simulations at 300 K (22 ns), simulations at 400 K (30 ns) and 500 K (30 ns) were performed for the two linear knottins EETI-II and lin-MCoTI and for the cyclic compound MCoTI-II. The curves in Figure 4, 5 and 8 suggest that the unfolding of MCoTI-II at 500 K is approximately similar to what is displayed by the linear compounds at 400 K, strongly suggesting a better resistance to temperature for the cyclic compound. This can be compared to recent reports on family 11 xylanase where MD simulations at 600 K, but not 300 K, revealed significant differences between mesophilic and thermophilic enzymes [51].

Nevertheless, all three studied compounds displayed high stabilities and no significant structure or flexibility differences between linear and cyclic compounds appeared in simulations at room temperature or in NMR experiments up to ~80°C. Rather, there are sequence differences that seem to be as or more important than circularization for structure and flexibility in standard conditions. For example, the sequence and type of the 22–25 *β*-turn differ in EETI-II and lin-MCoTI resulting in flexibility differences in this area (Figure 5), with EETI-II displaying lower flexibilities at 300 K and 500 K. Also, the addition of a charged residue (Lysine) in MCoTI-II and lin-MCoTI at position 13 induces transient salt bridging with Asp^20 ^thus perturbing the Asp^20^-O17 hydrogen bonding at the N-terminus of the 3–10 helix. This may be, at least partly, responsible for the lower flexibility at 300 K of the 3–10 helix in EETI-II in comparison to lin-MCoTI. More generally, the charged termini in linear compounds can in principle provide additional stabilizing electrostatic interactions when compared to cyclic analogs. As an example, a salt-bridge between the C-terminus and the side chain of an arginine in position 7 (Figure 1 numbering) has been reported in several linear squash inhibitors [43,52-54]. There is no such interaction here because both EETI-II and lin-MCoTI lack the Arg^7^, and, moreover, lin-MCoTI is uncharged at the C-terminus due to amidation.

Therefore circularization does not seem to be a key element of the squash inhibitors structure or flexibility in standard conditions. The cystine knot itself appears as the main factor responsible for the high stability and cyclization or sequence modifications seem to only induce marginal modifications. This does not hold however in high temperature simulations (500 K) where cyclization slowed the unfolding significantly, indicating that the cyclic squash inhibitor is more thermoresistant than linear counterparts. Despite the high flexibility of the head-to-tail linker in MCoTI-II, the cyclization is therefore likely to enhance resistance to strongly unfolding conditions.

### Comparison of cyclization in squash inhibitors and in cyclotides

Comparisons of solution structures suggested that the circularization in cyclotides provides a reduction of the flexibility, and this was supposed to be at the origin of the cancellation [23] or reduction [14] of the hemolytic activity in wild type or synthetic linear cyclotides. However it has been mentioned that the hemolytic activity could be related to hydrophobicity [55], and that the wild type linear cyclotide, violacin A, is less hydrophobic than other cyclotides [14]. It is thus unclear which of the linear or hydrophilic feature of violacin A is responsible for the reduced hemolytic activity. On the other hand, the linear synthetic cyclotides lacked few residues that could have impact on hemolytic activity [23,56], and to which extent linearization itself is involved in reduced activity remains to be determined. Moreover, stabilities of kalata B1 and acyclic permutants were shown to be very similar [24], suggesting that it is indeed the cystine knot rather than the circularization that provides most of the stability in cyclotides. It would be interesting to examine carefully the structural/functional differences between circular cyclotides and linear standard (hydrophobic) wild cyclotide when one is discovered.

Although the conclusions regarding the cyclization impact in the cyclotide family are roughly consistent with our results, it is worth noting that the head-to-tail linker in cyclotides is significantly different from the one in the cyclic squash inhibitor MCoTI-II. While the latter includes four glycines and no proline out of eight residues, the linker in most cyclotides includes one proline but only one glycine out of seven residues (see residues with numbers < 20 or > 100 in standardized alignments of cyclotides provided in the KNOTTIN database at knottin.cbs.cnrs.fr [2,3]). These sequence differences are probably sufficient to explain why this region is by far the most flexible part in MCoTI-II but not in the cyclotides since glycines are the most flexible residues and prolines the most rigid ones. One could then hypothesize that this well-structured linker is an integral part of the cyclotide structure possibly explaining why all cyclotides but one are circular. Conversely, the flexible linker in MCoTI-II is probably nothing more than an additional and very flexible loop, consistent with the fact that most squash inhibitors are linear. The presence of the linker in MCoTI-II could possibly provide extra contacts with the protease as well as protection to degradations by exoproteases or resistance to denaturing conditions.

### Biological role of knottin cyclization

The observations that the head-to-tail linker in MCoTI-II is the most flexible part of the molecule, and that linear squash inhibitors (e.g. EETI-II) can be as rigid as the circular MCoTI-II in standard conditions strongly suggests that the biological role of the circularization in squash inhibitors is not to reduce flexibility or to render the molecule more rigid. Nevertheless, better affinities for trypsin were achieved by circular squash inhibitors [Ki = 3.10^-11 ^M for MCoTI-II *vs*. 3.10^-10 ^M for lin-MCoTI and 8.10^-11 ^M for EETI-II [57]]. From the results in this work, it is unlikely that the improved affinity arises from conformational effects, either directly, or by reducing the flexibility of the free inhibitor, hence lowering the entropic loss due to binding. Our work rather suggests that the enhanced affinity of the circular compound could be due to direct binding of the head-to-tail linker with trypsin, as previously suggested by molecular modeling of the complex [13]. If this is the case, then the linker would have minimal impact in engineered squash inhibitor-based knottin variants, except in such particular cases where the linker bears a supplementary interaction site.

It is tempting to speculate that the biological role of the cyclization in squash inhibitors is for enzymatic rather than for thermodynamic reasons, and it has been suggested earlier that cyclization could prevent degradation by exoproteases [13,24]. The enzymatic stability of several knottins has been reported recently and both circular cyclotides and linear squash inhibitors were shown to display excellent resistance to proteases, except for the enzymes specific to the miniprotein, as e.g. serine proteases for squash inhibitors [4,24]. Even the naturally occurring linear cyclotide, violacin A, was shown to survive for 6 h in presence of trypsin or thermolysin reinforcing the idea that cyclization is not the main determinant for resistance to endoproteases [14]. Individual sequences may however display different enzymatic stabilities, and an example is provided by the human agouti-related protein that was shown to be proteolized more rapidly than squash inhibitors [4]. Interestingly, reduced cyclic kalata B1 has been shown to be more resistant to proteolysis than reduced linear conotoxin PVIIA [24]. This suggests that cyclization could also have some influence by slowing down enzymatic degradation of reduced knottins. Sensitivity of knottins to exoproteases, however, has not yet been systematically studied. Certainly, macrocyclic knottins will remain unaffected by exoproteases, but to which extent various linear knottins are sensitive to exoproteases remains to be determined. Both N- and C-terminal segments before and after the first and last well-structured half-cystines are rather short in many knottins, and it is unclear if these will be very sensitive to exoproteases. To our knowledge the only reported example comes from the linear cyclotide violacin A [14]. Violacin A was shown to be proteolysed by aminopeptidase M, which cleaved only the first two N-terminal residues. The third and fourth residues that precede the first cysteine of the knot were not cleaved, most likely because of their proximity to the cystine knot [14]. It can thus be hypothesized that many linear knottins with short N- and C-termini will not be easily degraded by exoproteases. Moreover, limited degradation of longer termini might not always be very deleterious since active residues are mostly located in inter-cysteine loops rather than in the termini. An example of this is provided by the minimized 34-residue agouti-related protein (AGRP) analog containing only the cystine knot domain, and which maintains the melanocortin receptor pharmacological profile of AGRP(87–132) [58]. Beside stability or protease resistance, cyclization was shown to facilitate *in vitro *oxidative folding of Kalata B1 that otherwise needs a hydrophobic environment to attain the native fold [21]. However it is worth noting that (i) *in vivo*, the formation of disulfide bridges is generally assumed to occur prior cyclization, thus bringing the termini close to each other for subsequent cyclization, and (ii) the phenomenon is specific to cyclotides since, in contrast, there is no need of cyclization or particular environment in the oxidative folding of squash inhibitors. The positive impact of cyclization on folding and/or resistance to denaturing conditions might well be important when membrane crossing is involved.

## Conclusion

We have shown that only small conformational differences are displayed by circular squash inhibitors and that linear compounds may display sufficient stabilities without the need for cyclization.

The cyclization observed in MCoTI-II, with no strong impact on thermodynamic or enzymatic stability, might therefore be an exceptional event rather than a general process in the squash family. One can even wonder if the increased affinity towards trypsin afforded by the cyclization (see above) is important for the plant itself since the affinities displayed by the linear compounds are already quite strong. Indeed, determining to which extent cyclization of squash inhibitors is a general process must await further large-scale studies in search of naturally occurring cyclized squash inhibitors in cucurbit seeds.

Protein circularization has been proposed as an interesting tool to stabilize engineered peptides in drug design studies [59-61]. Thanks to their small size, knottins have been shown to be readily accessible to chemical synthesis and routes to macrocyclic knottins are available [1,21,59,62,63]. Interestingly macrocyclic knottins were also recently shown to be accessible from bioengineering or biomimetic routes [22,27]. Nevertheless, peptide cyclization induces constraints on peptide synthesis and is expected to significantly lower the yields, especially when engineered sequences, that could be non-optimal for native-like folding, will be grafted onto the knottin scaffold. Thus the cost increase should be taken into account along with the potential benefit of circularization, if any, when considering circular knottin-based engineered molecules in drug design studies, since linear scaffolds with similar features are available, e.g. the squash inhibitor EETI-II. However, it is possible that stability can vary from one sequence to the other and that in particular cases circularization will be one good option to increase stability. But in many cases, the knottin scaffold itself and sequence optimization might be sufficient to insure high stabilities of linear compounds based on the squash inhibitor scaffold. An excellent example of knottin sequence optimization has been provided by incorporation of pairwise *β*-sheet stabilizing residues that improved folding and stability of the C-terminal knottin fold of the agouti signaling protein [64]. Nevertheless, the high temperature simulations we have performed suggest that the head-to-tail cyclization might help to stabilize squash inhibitors in strongly denaturing conditions and this may be important for membrane crossing. It is worth reminding also that when using the squash inhibitor scaffold in drug design, degradation by specific enzymes should generally be avoided. This can be easily achieved by mutating the protease sensitive site [44,57].

Finally, it can be concluded that the cystine knot is the main determinant for stability and for resistance to proteolysis of knottins. Then the specific sequence can help increasing both stability and resistance to proteolysis. Circularization can probably enhance resistance to strongly denaturing conditions and possibly facilitate *in vitro *folding in particular cases. Circularization may not be however a general prerequisite in knottin based drug design, especially when using the squash inhibitor scaffold.

## Methods

### Peptide synthesis

Wild type MCoTI-II was purified from seeds of the Gâc fruit (*Momordica cochinchinensis*) collected in Vietnam [13]. The linear variant lin-MCoTI was chemically synthesized using Fmoc solid phase peptide synthesis [57,65]. EETI-II was chemically synthesized as previously described [66].

### NMR experiments

Samples were prepared by dissolving peptides in either 90% H_2_O/10% ^2^H_2_O (v/v) or 100% ^2^H_2_O to a concentration of approximately 1.2 mM with the pH adjusted to 3.0 by addition of dilute HCl or NaOH. NMR spectra were recorded on a Bruker Avance-600 spectrometer equipped with a triple resonance inverse Cryoprobe with a single axis z gradient. Data were acquired at 12°C and 27°C, and TSP-d4 was used as an internal reference. All two-dimensional (2D) experiments, correlated spectroscopy (COSY), total correlated spectroscopy (TOCSY) and nuclear Overhauser effect spectroscopy (NOESY), were performed according to standard procedures [31] using quadrature detection in both dimensions with spectral widths of 6849.3 Hz in both dimensions. The carrier frequency was centred on the water signal and the solvent water resonance was suppressed by using the WATERGATE [67] method for experiments in H_2_O and by applying continuous low power irradiation during the relaxation delay and during the mixing time for NOESY spectra for experiments in ^2^H_2_O. The 2D spectra were obtained using 2048 or 4096 points for each t1 value, and 512 t1 experiments were acquired for COSY, TOCSY and NOESY experiments. TOCSY spectra were recorded with spin lock times of 30 and 60 ms. The mixing time was 150 and 300 ms in NOESY spectra. Spectra were processed using XWINNMR (Bruker). The t1 dimension was zero filled to 1024 points and *π*/3 or *π*/4 shifted sine bell functions were applied in t1 and t2 domains, respectively, prior to Fourier transform. ^3^J_NH-H*α*_coupling constants were measured on one-dimensional (1D) spectra. The exchange of amide protons with deuterium was studied at 12°C on samples lyophilized from H_2_O at pH 3.0 and dissolved in ^2^H_2_O. A series of 1D, TOCSY, and NOESY spectra were acquired over a 48-h period. ^1^H-^13^C hetero single quantum coherence spectroscopy (HSQC) and ^1^H-^13^C HSQC TOCSY spectra [68,69] were recorded on the samples in ^2^H_2_O. Spectral widths were 6849.3 Hz and 25000 Hz in the ^1^H and ^13^C dimensions respectively. 2048 data points were acquired with 512 t1 increments.

### Structure calculations

All calculations and analyses were performed on PC Linux boxes. The structures were displayed and analyzed using PyMOL (Warren L. DeLano "The PyMOL Molecular Graphics System." DeLano Scientific LLC, San Carlos, CA, USA. ). The NOE intensities were classified as strong, medium, and weak, and converted into distance constraints as previously described [26]. When necessary, the distance constraints were corrected for pseudoatoms [70]. Φ angles of residues with small or large ^3^J_HN-H*α *_coupling constants (< 4 Hz or > 8.5 Hz) were constrained into the -90° to -40° or -160° to -80° ranges, respectively. *χ*_1 _angles of residues for which stereospecific attribution of the *β *protons could be achieved were constrained in the corresponding range. Disulfide bridges were imposed through distance constraints of 2.0–2.1, 3.0–3.1, and 3.75–3.95 Å on Si-Sj, Si-C*β*j and Sj-C*β*i, and C*β*i-C*β*j distances, respectively. No hydrogen bond was imposed. Three hundred 3D structures were obtained as previously [26] from the distance and angle restraints using the torsion angle molecular dynamics method available in the CYANA program [71]. The fifty structures with the lowest violation of the target function were submitted to molecular mechanics energy refinement with the SANDER module of the AMBER 8 program [40], using the ff03 force field [72] and the GB/SA implicit solvation scheme [72]. During the restrained molecular dynamics runs the covalent bond lengths were kept constant by applying the SHAKE algorithm [73] allowing a 2 fs time step to be used. Distance and angle NMR restraints were applied using square bottom wells with parabolic sides. The sides become linear for large deviations [26]. When no stereospecific assignment could be achieved for methyl or methylene protons, an < r^-6 ^> ^-1/6 ^averaging scheme was used instead of pseudo-atoms. No constraints were applied to the disulfide bridges. Five thousand cycles of restrained energy minimization were first carried out followed by a 100-ps long simulated annealing procedure in which the temperature was raised to 900 K for 40 ps then gradually lowered to 300 K. During this stage, the force constant for the NMR distance and dihedral restraints were gradually increased from 3 to 30 kcal.mol^-1^.Å^-2 ^or kcal.mol^-1^.rad^-2^.

### Thermal unfolding

The thermal unfolding was monitored using 1D NMR data obtained on samples in ^2^H_2_O previously used for exchange experiments. The temperature within the NMR samples was calibrated with ethylene glycol and varied between 10°C and 80°C. Chemical shifts for fully unfolded species *δ*_U _could not be attained experimentally and were taken as random coil chemical shifts of the corresponding protons [74-76]. Since we could not precisely determine chemical shifts for fully folded species *δ*_F_, these were obtained from fitting experimental *δ*(T), the chemical shift at temperature T, to a simple two-state unfolding mechanism [34,41] using Kaleidagraph (Synergy Software, Reading):

*δ*(T) = *α δ*_F _+ (1-*α*) *δ*_U_

where *α *= 1/1 + *e*^-Δ*G*|*RT*^

Then the two-state equilibrium constant for thermal unfolding,

$$K_{U}^{thermal}=f_{U}/(1-f_{U})=\left(\delta_{F}-\delta \left(T\right)\right)/\left(\delta \left(T\right)-\delta_{U}\right)$$

and

$$\Delta G_{U}^{thermal}=-RT\ln K_{U}^{thermal}$$

was determined at each experimental temperature (f_U_, fraction unfolded). The Tm values for reversible thermal unfolding of the peptides were calculated by linear fitting of $\Delta G_{U}^{thermal}$ versus T for each proton.

The statistical error on experimental chemical shifts was estimated to ± 0.02 ppm. To obtain a rough estimate of the corresponding error on the calculated thermodynamics parameters, the above calculations were repeated 100 times with chemical shifts randomly picked in the range of x ± 0.02 ppm, where x is the experimentally determined value. From the resulting distributions of parameters, mean values of Tm, and associated standard deviations were calculated and are reported in Table 5.

### Molecular dynamics simulations

Molecular dynamics simulations were carried out on an AMD Opteron cluster using the PGI compilers (The Portland Group, Inc., Portland, USA) and the AMBER 8.0 program [40]. The starting models were immersed into a truncated octahedron of TIP3P explicit water models [77], with minimal distances of 15 Å between any protein atom and the box boundaries. Periodic boundary conditions were imposed and the total charge of the system was compensated for by using a neutralizing plasma. Lennard-Jones and electrostatic interactions were calculated using the Particle-mesh Ewald (PME) summation scheme [78], with a cut-off of 8 Å for the separation of the direct and reciprocal space summation.

Water molecules were first energy minimized while restraining the protein atoms. Then, the whole system was equilibrated for 0.5 ns at the target temperature and 1 bar using the weak coupling algorithm (temperature and pressure relaxation times = 2 ps) [79]. For production runs, the temperature was regulated using the Langevin dynamics with a collision frequency of 3 ps^-1^, and bonds involving hydrogen atoms were constrained using the SHAKE algorithm [73]. The conformations were stored every 1 ps, and the trajectories were analyzed with the Ptraj program of the Amber 8.0 suite. Room temperature molecular dynamics simulations were performed at 300 K for 22 ns. Unfolding simulations were performed at higher temperatures (400 K and 500 K) for 30 ns. The structural criteria used to monitor protein unfolding were the RMSD and a nativeness score, the Q-score. The Q-score was computed using the MMTSB tool available at . It is calculated using a Gaussian function of the inter-residue C*α *distance centered at zero with standard deviation of |*j-i*|^0.15 ^and normalized by the number of non-bonded-contacts [42]

## Authors' contributions

AH participated in the design of the study, carried out the NMR study and helped to draft the manuscript. OA carried out the chemical synthesis of lin-MCoTI. DLN carried out the chemical synthesis of EETI-II and helped to draft the manuscript. UD supervised the synthesis of lin-MCoTI. JFH participated in the design of the study and drafted the manuscript. JG implemented computer tools, carried out simulations and helped to draft the manuscript. HK conceived the study and participated in its design and coordination. LC conceived the study, participated in its design and coordination, carried out simulations, and drafted the manuscript.

## Supplementary Material

Additional file 1 **NMR spectra of lin-MCoTI**. 600 MHz spectra at 300 K in 90% H_2_O/10% ^2^H_2_O at pH 3.0 (Top) TOCSY spectrum. Amino acid spin systems are labeled. (Bottom) Fingerprint region of the NOESY spectrum showing sequential connectivities between the residues for the 10–21 and 23–34 regions. Click here for file

Additional file 2 **Chemical shifts in ppm for lin-MCoTI**. ^1^H chemical shifts (12 and 27°C) and ^13^C chemical shifts (27°C) in ppm for lin-MCoTI. Values were measured at pH 3.0 in H_2_O/D_2_O (^1^H) or in D_2_O (^13^C) relative to TSP-d4 as internal reference. The numbering starts at 1 for the first residue and does not follow the numbering used in the text and shown in Figure 1. Click here for file
