# Supplementary figures and images for: Knottin cyclization: impact on structure and dynamics
Source: BMC Struct Biol. 2008 Dec 12;8:54. doi: 10.1186/1472-6807-8-54 (PMC2659701; doi:10.1186/1472-6807-8-54)

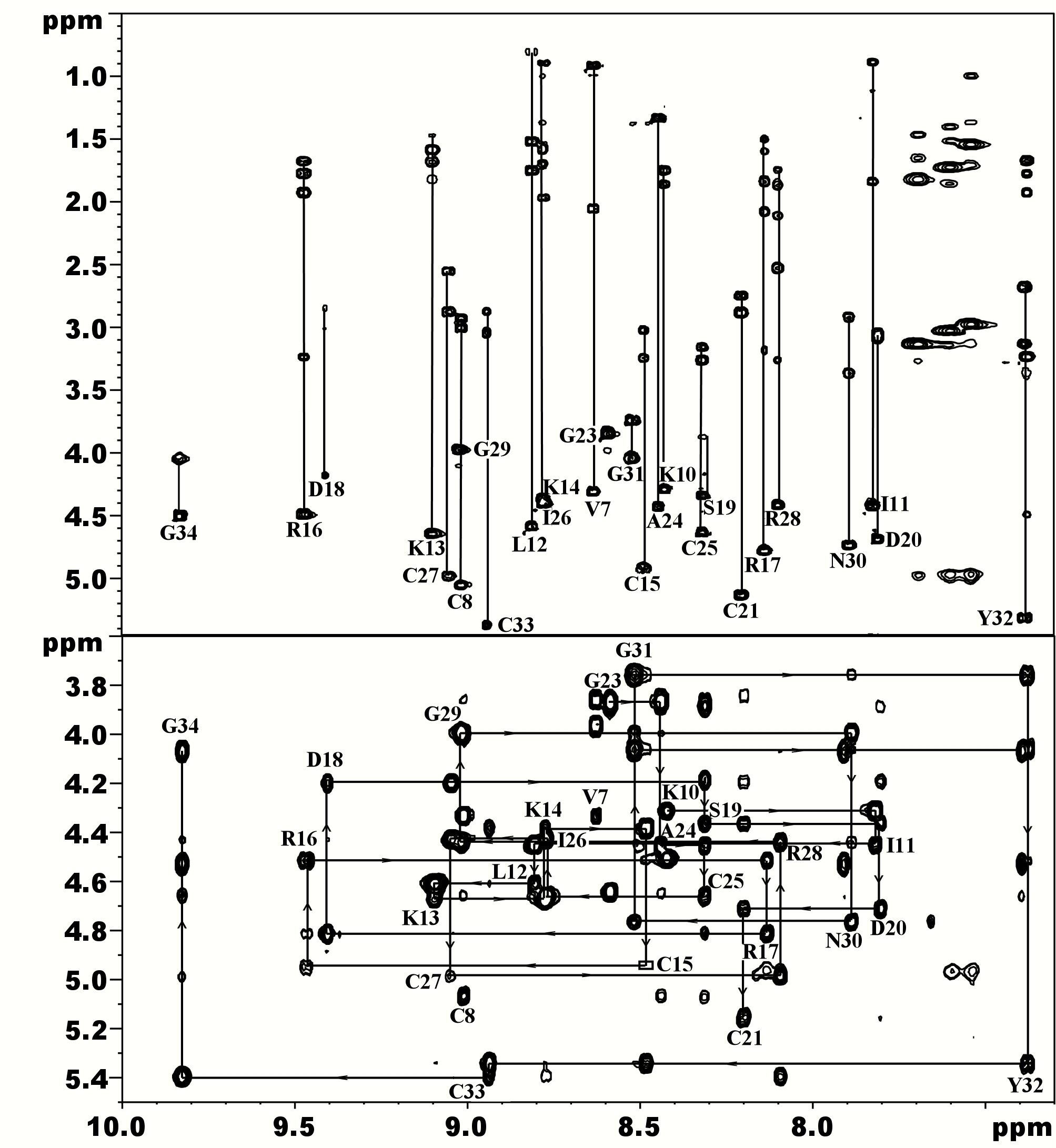

Supplement: Additional file 1 — NMR spectra of lin-MCoTI. 600 MHz spectra at 300 K in 90% H2O/10% 2H2O at pH 3.0 (Top) TOCSY spectrum. Amino acid spin systems are labeled. (Bottom) Fingerprint region of the NOESY spectrum showing sequential connectivities between the residues for the 10–21 and 23–34 regions. [file 1472-6807-8-54-S1.png]
