# Supplementary material for: Knottin cyclization: impact on structure and dynamics
Source: BMC Struct Biol. 2008 Dec 12;8:54. doi: 10.1186/1472-6807-8-54 (PMC2659701; doi:10.1186/1472-6807-8-54)
Supplement: Additional file 2 — Chemical shifts in ppm for lin-MCoTI. 1H chemical shifts (12 and 27°C) and 13C chemical shifts (27°C) in ppm for lin-MCoTI. Values were measured at pH 3.0 in H2O/D2O (1H) or in D2O (13C) relative to TSP-d4 as internal reference. The numbering starts at 1 for the first residue and does not follow the numbering used in the text and shown in Figure 1. [file 1472-6807-8-54-S2.pdf]

Additional file 2 : Chemical shifts in ppm for lin-MCoTI

Table 1: ^{1}H chemical shifts in ppm for lin-MCoTI at 12 °C

^{1}H chemical shifts in ppm for lin-MCoTI. Values were measured ± 0.02 ppm relative to TSP-d4 as internal reference. Conditions used were 12 °C, pH 3.0, H_2O/D_2O (9:1 by vol.). The numbering starts at 1 for the first residue and does not follow the numbering used in the paper and shown in Figure 1.

N°	Residue	NH	Hα	Hβ	Others
1	Gly		3.96-3.86		
2	Val	8.63	4.33	2.07	γCH_3 0.93
3	Cys	9.01	5.05	3.01-2.93	
4	Pro		4.50	2.36-1.97	γCH_2 2.07-1.97, δCH_2 3.99-3.85
5	Lys	8.42	4.30	1.87-1.77	γCH_2 153-1.41, δCH_2 1.75, εCH_2 3.0,5 ζNH_2 7.60
6	Ile	7.82	4.44	1.85	γCH_2 1.44-1.13, γCH_3 0.90, δCH_3 0.90
7	Leu	8.81	4.60	1.77-1.54	γCH 1.54, δCH_3 0.84-0.80
8	Lys	9.10	4.66	1.69-1.60	γCH_2 1.64-1.47, δCH_2 1.83, εCH_2, 3.16 ζNH_2 7.69
9	Lys	8.78	4.37	1.71-1.59	γCH_2 1.37-1.00, δCH_2 1.56, εCH_2 2.99, ζNH_2 7.54
10	Cys	8.48	4.93	3.26-3.04	
11	Arg	9.47	4.50	1.94-1.78	γCH_2 1.68 δCH_2 3.25 εNH 7.38
12	Arg	8.13	4.80	2.10-1.85	γCH_2 1.60-1.50, δCH_2 3.22-3.18, εNH 7.24
13	Asp	9.40	4.19	3.02-2.85	
14	Ser	8.31	4.36	4.17-3.88	
15	Asp	7.80	4.70	3.10-3.04	
16	Cys	8.20	5.14	2.90-2.75	
17	Pro		4.63	2.36-1.98	γCH_2 2.16-2.09, δCH2 3.83-3.45
18	Gly	8.59	3.86		
19	Ala	8.44	4.44	1.34	
20	Cys	8.31	4.65	3.27-3.16	
21	Ile	8.77	4.42	1.98	γCH_2 1.27-1.23, γCH_3 0.91, δCH_3 0.88
22	Cys	9.04	4.97	2.88-2.56	
23	Arg	8.10	4.43	2.55-2.12	γCH_2 1.86-1.75, δCH_2 3.27, εNH 7.06
24	Gly	9.02	3.99		
25	Asn	7.89	4.75	3.38-2.93	δNH_2 7.66-6.67
26	Gly	8.52	4.06-3.76		
27	Tyr	7.38	5.32	3.14-2.69	2,6H 6.94, 3,5H 6.76
28	Cys	8.94	5.38	3.06-2.88	
29	Gly	9.82	4.52-4.06		NH_2 7.91-7.40

Table 2: ^{1}H chemical shifts in ppm for lin-MCoTI at 27 °C

^{1}H chemical shifts in ppm for lin-MCoTI. Values were measured ± 0.02 ppm relative to TSP-d4 as internal reference. Conditions used were 27 °C, pH 3.0, H_2O/D_2O (9:1 by vol.). The numbering starts at 1 for the first residue and does not follow the numbering used in the paper and shown in Figure 1.

N°	Residue	NH	Hα	Hβ	Others
1	Gly		3.94-3.83		
2	Val	8.52	4.30	2.05	γCH_3 0.91
3	Cys	8.87	5.04	3.00-2.92	
4	Pro		4.47	2.37-1.94	γCH_2 2.06-1.94, δCH_2 3.97-3.83
5	Lys	8.30	4.29	1.86-1.74	γCH_2 150-1.40, δCH_2 1.72, εCH_2 3.03, ζNH_2 7.53
6	Ile	7.73	4.42	1.84	γCH_2 1.40-1.10, γCH_3 0.88, δCH_3 0.88
7	Leu	8.69	4.57	1.74-1.51	γCH 1.51, δCH_3 0.82-0.78
8	Lys	8.98	4.63	1.66-1.61	γCH_2 1.64-1.46, δCH_2 1.81, εCH_2 3.14 ζNH_2 7.63
9	Lys	8.66	4.38	1.70-1.59	γCH_2 1.36-1.00, δCH_2 1.54, εCH_2 2.97, ζNH_2 7.47
10	Cys	8.44	4.92	3.24-3.02	
11	Arg	9.41	4.48	1.93-1.77	γCH_2 1.67, δCH_2 3.23, εNH 7.32
12	Arg	8.09	4.77	2.08-1.83	γCH_2 1.60-1.50, δCH_2 3.19, εNH 7.18
13	Asp	9.28	4.17	3.00-2.83	
14	Ser	8.17	4.33	4.14-3.87	
15	Asp	7.75	4.67	3.07-3.01	
16	Cys	8.13	5.11	2.89-2.75	
17	Pro		4.61	2.35-1.96	γCH_2 2.14-2.07, δCH_2 3.80-3.44
18	Gly	8.46	3.83		
19	Ala	8.32	4.42	1.33	
20	Cys	8.25	4.64	3.25-3.16	
21	Ile	8.71	4.40	1.96	γCH_2 1.24-1.20, γCH_3 0.88, δCH_3 0.85
22	Cys	9.00	4.93	2.86-2.55	
23	Arg	8.05	4.41	2.50-2.10	γCH_2 1.86-1.72, δCH_2 3.25, εNH 7.00
24	Gly	8.89	3.96		
25	Asn	7.84	4.73	3.36-2.91	δNH_2 7.57-6.60
26	Gly	8.47	4.03-3.73		
27	Tyr	7.34	5.30	3.12-2.67	2,6H 6.92, 3,5H 6.74
28	Cys	8.86	5.34	3.05-2.86	
29	Gly	9.76	4.49-4.03		NH_2 7.81-7.29

Table 3: ^{13}C chemical shifts in ppm for lin-MCoTI at 27 °C

^{13}C chemical shifts in ppm for lin-MCoTI. Values were measured ± 0.1 ppm relative to TSP-d4 as internal reference. Conditions used were 27 °C, pH 3.0, D_2O. The numbering starts at 1 for the first residue and does not follow the numbering used in the paper and shown in Figure 1.

N°	Residue	Cα	Cβ	Others
1	Gly	42.8		
2	Val	61.1	33.6	γCH_3 20.8-19.9
3	Cys	52.5	40.6	
4	Pro	62.5	32.4	γCH_2 26.9, δCH_2 51.2
5	Lys	56.3	30.8	γCH_2 24.7, δCH_2 28.9, εCH_2 41.8
6	Ile	59.4	41.1	γCH_2 26.7, $\gamma CH_3$17.2, δCH_3 13.9
7	Leu	54.1	41.6	γCH 27.1, δCH_3 24.3-24.2
8	Lys	55.4	36.0	γCH_2 25.0, δCH_2 29.0, εCH_2 42.3
9	Lys	56.2	33.2	γCH_2 25.4, δCH_2 28.9, εCH_2 41.6
10	Cys	53.6	48.0	
11	Arg	55.9	32.1	γCH_2 27.0, δCH_2 42.8
12	Arg	53.5	32.9	γCH_2 25.6, δCH_2 43.2
13	Asp	58.9	39.4	
14	Ser	59.8	62.0	
15	Asp	55.6	42.2	
16	Cys	51.6	40.0	
17	Pro	62.0	32.2	γCH_2 27.3, δCH_2 50.0
18	Gly	47.1		
19	Ala	51.8	18.2	
20	Cys	55.0	44.4	
21	Ile	59.0	41.0	γCH_2 26.0, γCH_3 18.9, δCH_3 13.4
22	Cys	55.1	38.0	
23	Arg	56.1	30.8	γCH_2 27.6, δCH_2 43.1
24	Gly	46.7		
25	Asn	52.1	36.8	
26	Gly	46.0		
27	Tyr	56.8	41.4	2,6C 131.0, 3,5C 115.4
28	Cys	55.1	40.6	
29	Gly	45.9		
